# Multiplatform Investigation of Plasma and Tissue Lipid Signatures of Breast Cancer Using Mass Spectrometry Tools

**DOI:** 10.3390/ijms21103611

**Published:** 2020-05-20

**Authors:** Alex Ap. Rosini Silva, Marcella R. Cardoso, Luciana Montes Rezende, John Q. Lin, Fernando Guimaraes, Geisilene R. Paiva Silva, Michael Murgu, Denise Gonçalves Priolli, Marcos N. Eberlin, Alessandra Tata, Livia S. Eberlin, Sophie F. M. Derchain, Andreia M. Porcari

**Affiliations:** 1 Postgraduate Program of Health Sciences, São Francisco University, Bragança Paulista SP 12916-900, Brazil; alexrosinisilva@hotmail.com (A.A.R.S.); denise.priolli@usf.edu.br (D.G.P.); 2 Department of Gynecological and Breast Oncology, Women’s Hospital (CAISM), Faculty of Medical Sciences, State University of Campinas (UNICAMP), Campinas SP 13083-881, Brazil; macardoso86@hotmail.com (M.R.C.); luciana_mrezende@hotmail.com (L.M.R.); fernando@caism.unicamp.br (F.G.); derchain@fcm.unicamp.br (S.F.M.D.); 3 Department of Chemistry, The University of Texas at Austin, Austin, TX 78712, USA; john@jqlin.com (J.Q.L.); liviase@utexas.edu (L.S.E.); 4 Laboratory of Molecular and Investigative Pathology—LAPE, Women’s Hospital (CAISM), Faculty of Medical Sciences, State University of Campinas (UNICAMP), Campinas SP 13083-881, Brazil; geisi@unicamp.br; 5 Waters Corporation, São Paulo, SP 13083-970, Brazil; Michael_Murgu@waters.com; 6 School of Engineering, Mackenzie Presbyterian University, São Paulo SP 01302-907, Brazil; mneberlin@gmail.com; 7 Laboratorio di Chimica Sperimentale, Istituto Zooprofilattico Sperimentale delle Venezie, Viale Fiume 78, 36100 Vicenza, Italy; atata@izsvenezie.it

**Keywords:** breast cancer, liquid chromatography-mass spectrometry, desorption-electrospray-ionization—mass spectrometry imaging, lipidomics, plasma, tumor tissue

## Abstract

Plasma and tissue from breast cancer patients are valuable for diagnostic/prognostic purposes and are accessible by multiple mass spectrometry (MS) tools. Liquid chromatography-mass spectrometry (LC-MS) and ambient mass spectrometry imaging (MSI) were shown to be robust and reproducible technologies for breast cancer diagnosis. Here, we investigated whether there is a correspondence between lipid cancer features observed by desorption electrospray ionization (DESI)-MSI in tissue and those detected by LC-MS in plasma samples. The study included 28 tissues and 20 plasma samples from 24 women with ductal breast carcinomas of both special and no special type (NST) along with 22 plasma samples from healthy women. The comparison of plasma and tissue lipid signatures revealed that each one of the studied matrices (i.e., blood or tumor) has its own specific molecular signature and the full interposition of their discriminant ions is not possible. This comparison also revealed that the molecular indicators of tissue injury, characteristic of the breast cancer tissue profile obtained by DESI-MSI, do not persist as cancer discriminators in peripheral blood even though some of them could be found in plasma samples.

## 1. Introduction

The use of omics technologies for breast cancer investigations has impacted our understanding of how the molecular alterations, at multiple levels, lead to carcinogenesis [1]. Although no significant clinical gains have been conquered yet, metabolomics and lipidomics studies have led to the discovery of an increasing number of molecules suggested as possible biomarkers for breast cancer [2]. Robust biomarkers, able to improve diagnosis and prognosis, are highly attractive and multiple analytical platforms may act as complementary tools in the search for them. Powerful features such as superior sensitivity, simultaneous detection of multiple compounds, ability to employ small sample volumes, and ease of coupling to chromatographic techniques have allowed mass spectrometry (MS) to occupy an increasingly prominent place in clinical diagnosis [3].

In the field of clinical diagnosis, liquid chromatography-mass spectrometry (LC-MS) has been extensively exploited for blood analysis of breast cancer patients to achieve early diagnosis [4,5,6,7,8,9,10,11], metabolic reprogramming [12,13], cancer typing or staging [14] and therapeutic intervention response [15], as recently reviewed [16,17,18].

While LC-MS is very suitable for biofluid analysis, MS imaging (MSI) is another outstanding MS technique that has gained attention for direct tissue analysis [19,20]. MSI provides comprehensive information on the distribution of the molecules directly over the surface of samples. MS-based tissue imaging applied to clinical research has been accelerated by the development of ambient ionization MS, that allows the samples to be analyzed with minimal or no sample preparation, in an open environment and at atmospheric pressure [21]. Other features such as the shorter time and the non-destructive nature of the analysis have motivated investigations using ambient MSI in the intra-operative environment [22,23]. Ambient MSI is being increasingly used for metabolomics investigations with a particular interest in lipids since these molecules are abundant over cell membranes and easily ionized under ambient conditions [24,25,26,27]. Desorption-electrospray-ionization-MSI (DESI-MSI) is one of the most prominent ambient MSI techniques and has recently demonstrated its value in the study of breast cancer. DESI-MSI has proved to be a robust and reproducible technology for rapid breast-cancer-tissue diagnosis and margin analysis [28,29,30,31,32], differentiation of necrotic areas in breast cancer [33], and identification of in situ and invasive area of breast carcinoma across the different molecular subtypes [34].

Using DESI-MSI, our lab recently carried out a multicenter investigation [28] for a diverse population set comprising different patient races from different countries and built statistical classifiers able to discriminate no special type (NST) invasive ductal carcinoma (IDC) tissue from normal tissue. The model was also able to predict the status of estrogen receptor (ER), progesterone receptor (PR), and the resulting hormone receptor status (HR) in IDC. Overall, DESI-MSI enabled us to discriminate distinct histological regions over the tissue, and molecular signatures specific of carcinoma cells were selected from the surrounding stroma, vessels, adipose tissue, and normal glands, besides generating a robust predictive statistical model for breast cancer diagnosis. In the present study, we wondered whether a correspondence exists between lipid cancer signatures observed by DESI-MSI of cancer tissue and those detected by LC-MS in plasma.

To answer this question, we first carried out an accurate literature search and realized that only a few studies correlated the lipid profile of tissues obtained by DESI-MSI to those obtained by LC-MS [35,36,37,38]. Abbassi-Ghadi et al. compared DESI-MS lipid profiling of tissue to LC-MS lipid profiling of the same excised and extracted tissue [39]. The authors demonstrated that the lipid profile observed by DESI-MS is congruent to that obtained by LC-MS, which confirmed the role of DESI-MS for lipid profiling as a stand-alone technique. To the best of our knowledge, a comparison between the cancerous biomarkers recovered by LC-MS in circulating plasma and those detected through direct tissue analysis by DESI-MS has not been made yet.

Therefore, in this pilot study, the lipid profile of plasma obtained by LC-MS was correlated to that revealed by DESI-MSI of core biopsies and excised tumors of Brazilian women with breast cancer. Both special and NST types of carcinoma were evaluated using the model described by our group [28] as a complementary validation set. To determine putative plasma biomarkers, LC-MS analysis of plasma from cancer women was also compared to a control group.

## 2. Results

### 2.1. Molecular Imaging of Breast Tissues by DESI-MSI

**Figure 1:**
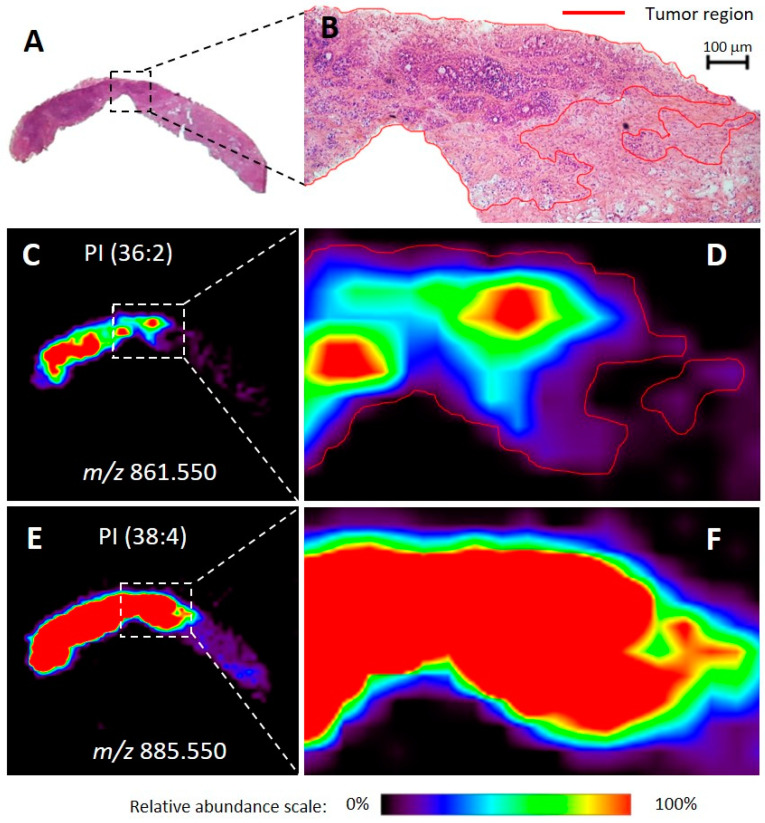
Optical images of hematoxylin & eosin (H&E) stained slides and representative ion images for a biopsied tissue sample diagnosed as invasive apocrine carcinoma of the breast with a signet ring. The entire tissue section is shown in (**A**) and the optical magnification (40×) of a region is shown in (**B**), with red-marked tumor areas. The representation of the relative abundance of a tumor discriminatory ion of mass-to-charge ratio (*m/z*) 861.550 is shown in (**C**). Zoomed in and outlined in red, (**D**) shows a comparative perspective with the H&E image above (**B**). The representation of the relative abundance of a non-discriminatory ion of m/z 885.550 is shown in (**E**). Also, zoomed-in, (**F**) shows a comparative perspective with images D and B above. Areas of red intensity within the ion images represent the highest (100%) and black the lowest (0%) relative abundance. PI: glycerophosphoinositol. Lipid species are described by the numbers of fatty acid chain carbons and double bonds.

Twenty-eight human breast tissue samples were analyzed using DESI-MSI. These samples included 11 special type samples and 17 NST samples obtained from core biopsy fragments or surgical specimens (Supplementary Table S1). DESI-MSI enabled visualization of histological features within ductal carcinoma (DC) samples (i.e., fibrosis, stroma, normal glands, IDC regions, in situ DC, vessels, etc.), as previously reported [28,29,30]. The molecular images generated could then be correlated with optical images of the hematoxylin & eosin (H&E) stained tissue sections (Figure 1). Indeed, molecular images of the glycerophosphoinositol (PI) and fatty acid (FA) ions could serve as markers for the tumor areas over the tissue. Tumor regions spatially correlated with the distribution of PI(36:2) and FA(20:4). Other lipids such as PI(38:4) showed no specificity around stroma, fibrosis, normal adjacent tissue, or tumor regions and were found over the entire tissue section. Molecular images of both NST and special type samples correctly correlated with the optical images. Figure 1 shows the good correspondence between the optical microscopy images and the ion images of a biopsy fragment of a special type tumor, exemplifying that these molecules are characteristic of cancer, independently of the type of breast cancer and the type of tumor sampling.

**Figure 2:**
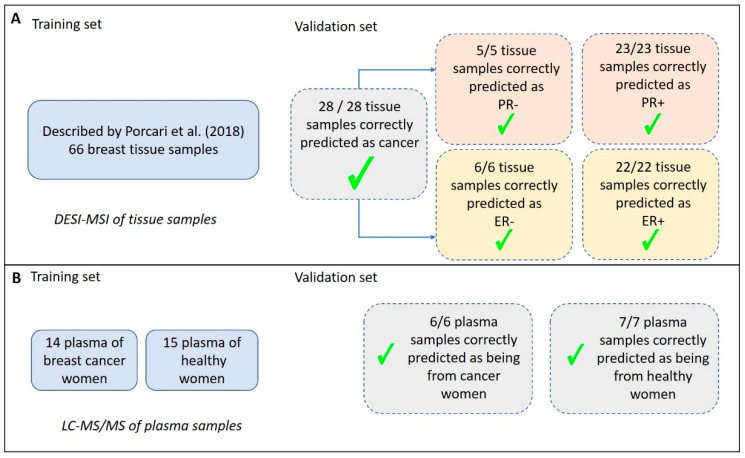
Summary of the classification predictions of breast carcinoma tissue and plasma samples. (**A**) Results obtained for tissue analysis using DESI-MSI (Desorption-Electrospray-Ionization—Mass Spectrometry Imaging). A previously validated model for classification of samples was described by Porcari et al. [28], with 66 breast cancer samples compared to normal breast tissue, and it was used here as a test set. In the validation set, all the NST (no special type) and special type tissue samples were correctly classified as being cancer and as having +/- Progesterone Receptor (PR) and +/- Estrogen Receptor (ER). (**B**) Describes the results obtained for plasma analysis using LC-MS/MS (Liquid Chromatography—tandem Mass Spectrometry). The test set was composed of 29 plasma samples and resulted in average accuracies of 99.8% (positive ion mode) and 99.2% (negative ion mode) based on 100 cross-validations. In the validation set, including 30% of the samples, all the plasma samples were correctly classified as being cancer or not.

The set of samples comprising both NST and special type carcinomas, which were collected and analyzed in Brazil, was later submitted to a blind classification test performed in the USA, using the predictive models of NST-IDC built for lipid DESI-MSI data described by our group [28]. All the 28 breast samples, including special type tumors, were correctly classified according to their cancer status (as being cancer or normal samples). The predictive model was also able to define their ER and PR status, as being positive or negative (Figure 2).

The results of the classification test showed that all the 28 samples were correctly predicted as being cancer samples. From these, 6 samples were correctly classified as ER-negative, while 22 samples were classified (also correctly) as ER-positive. Similarly, 5 samples were correctly classified as PR negative, whereas 23 samples were classified (also correctly) as PR positive. The results presented for cancer status prediction, ER and PR status classification showed, therefore, a sensitivity of 100% and specificity of 100%, in a per-patient analysis, regardless of the type of IDC samples (NST- or special type-IDC).

### 2.2. Analysis of Plasma by LC-MS/MS

**Figure 3:**
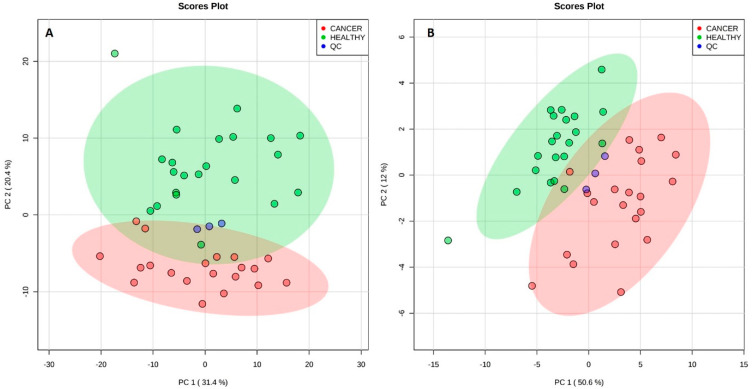
Principal component analysis (PCA) scores plot for plasma analysis in positive ion mode (**A**) and in negative ion mode (**B**). Segregation was observed for both modes between cancer and healthy individuals. Quality control (QC) samples (pool of all the samples) are also plotted. The explained amount of the total variance of the full data set is shown for each principal component (PC1-2).

The sum of 42 plasma samples obtained from 22 healthy volunteers (control) and 20 breast cancer women (case) had their organic extracts analyzed by LC-MS/MS. A total of 1434 compounds were detected in positive ion mode, whereas 1480 compounds were detected in negative ion mode. From these, 892 compounds were automatically identified using Progenesis QI based on accurate mass, isotope similarity, and MS^E^ experiments. In this untargeted approach, the relative intensities of the ions, normalized to the total ion current (TIC), were considered to detect changes among the groups. Examples of the chromatograms, raw spectra, and feature identification are shown in the Supplementary Figures S1–S3, for the positive and negative ion modes. Using all the detected compounds, unsupervised multivariate analysis was performed for data from both the positive and negative ion modes. Figure 3 shows the Principal Component Analysis (PCA) score plots for both ionization modes. Although some overlap of the 95% confidence level ellipses occurs in both plots, a clear tendency of segregation of cancerous and healthy individuals by PCA is observed.

Based on the plasma composition of 70% of the samples (training set) from the two ionization modes, two classification models were built using the Supporting Vector Machine (SVM) algorithm and they were tested for their ability to classify unknown samples (30%) comprising the validation set. SVM models were also used to point out which of the detected molecules would contribute more significantly to such differentiation. To build the classification models, 8 predictive molecular features were selected for positive ion mode, and 9 for negative ion mode. Table 1 shows the selected molecular features together with their possible assignments. Features selection was based on their area under the curve (AUC) value for their individual receiver operating-characteristic (ROC) curves, a parameter that indicates their ability to distinguish between healthy and cancer plasma. The overall prediction power of these two models was estimated based on the AUC of ROC plots obtained from the combination of the AUC of all the selected features. Both models presented the maximum AUC value (1.00), indicating how well the set parameters could distinguish between case and control groups. As Figure 2 summarizes, the resulting SVM models were applied to classify the validation set. Both the SVM models correctly classified 7 out of 7 healthy samples and 6 out of 6 cancer samples, resulting in maximum positive and negative predictive values (PPV/NPV), specificities of 100%, and sensitivity of 100% in a per-patient analysis. The medium probability of correct classification found for the validation set is a value that indicates the probability of each specific sample in the data set to be classified as being part of a determined class. In the present study, this value was found as 93.6% for positive ion mode SVM model and 92.7% for negative ion mode SVM model. If these medium probabilities were close to 0.5 then the model would have insufficient discriminatory power and should not be used for predictions [40].

### 2.3. Correspondence of Biomarkers Between Tissue-DESI-MSI and Plasma-LC-MS

To verify the presence of tissue biomarkers among the metabolites detected in plasma, the set of tissue biomarkers reported by Porcari et al. [28] and used to classify the tissue samples of this study were sought among the 892 compounds which had automatically been identified in plasma samples. From the list of 31 compounds previously reported as discriminators of breast cancer by DESI-MSI, 17 were detected among the plasma metabolites. Table 2 summarizes the list of DESI-MSI tissue biomarkers and their occurrence in plasma samples.

**Figure 4:**
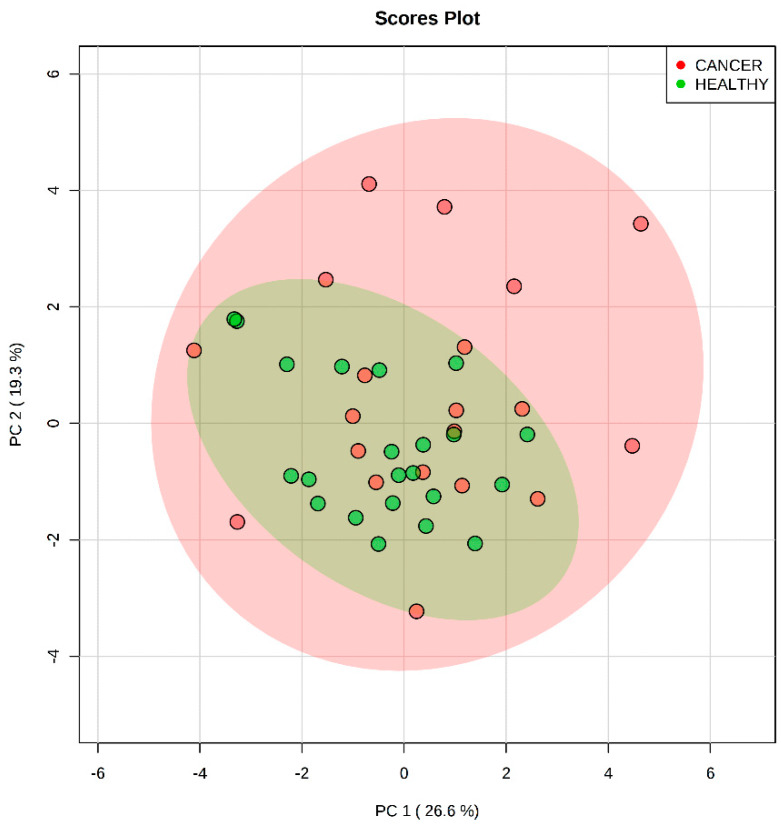
Principal component analysis scores-plot of the differentiation of plasma samples analyzed by LC-MS/MS (Liquid Chromatography—tandem mass spectrometry) using the molecules found in both plasma and tissue by DESI-MSI (Desorption-Electrospray-Ionization—Mass Spectrometry Imaging) [28]. The set of selected features does not enable group differentiation. Principal component (PC) 1 explains 33.3% of the total variance of the full data set, whereas PC2 explains 25.7%.

The set of 17 mutual molecules (Table 2) was used to build a PCA model aimed at distinguishing between the case and control plasma samples (Figure 4). The abundances of these molecules in plasma for negative ion mode were considered. No differentiation of the groups was achieved. An SVM model was also built and showed a near to diagonal line for the ROC curve (Figure S4), confirming the poor differentiation power of this set of molecules.

## 3. Discussion

In our study, multiple MS techniques were used to establish a comprehensive diagnostic workflow for the different sample types collected from breast patients (i.e., plasma, core biopsy, and surgical specimen). DESI-MSI correctly assigned tumor regions both in pre-surgical and post-surgical tissue slides, in excellent agreement with the pathologist’s evaluation. Plasma analysis through LC-MS/MS revealed putative lipid biomarkers for both positive and negative ionization modes and our models showed excellent sensitivities and accuracy in a per-patient analysis. The correlation of plasma and tissue lipids showed that some of the tissue biomarkers were also detected in plasma samples, although these molecules were not found as significant contributors for plasma differentiation among healthy and breast cancer groups.

### 3.1. Molecular Imaging of Breast Tissues by DESI-MSI

In the present study, we analyzed biopsies and surgical specimens of breast cancer patients including NST and special type tumors, to verify whether our model, previously built based solely on NST samples, would also be able to correctly classify special type tumors.

Our study submitted DESI-MSI data of 28 tissue samples, including 12 samples from special type tumors, to our pre-generated classification model, again with an inter-laboratory approach. Remarkably, 100% of specificity, sensitivity, and accuracy were achieved for this new validation set in a per-patient analysis, even when special tumors were considered. These results emphasize that a single Lasso model built from DESI-MSI can classify inter-laboratory independent sample sets and that the putative biomarkers pointed out by this model for IDC breast tumors are robust enough to differentiate special tumor types.

### 3.2. Analysis of Plasma by LC-MS/MS

Healthy and cancerous plasmas, as previously reported [8,41,42], have a distinct lipid profile. These differences were able to discriminate these groups both by unsupervised and supervised multivariate analysis. Using the support vector machine (SVM), a multivariate classification method that applies a non-parametric machine learning technique, two sensitive and accurate models for plasma sample differentiation were built [40,43]. These models (built for the positive and negative ionization modes) are independent and complementary to one another. To deepen the understanding of metabolic pathways involved in breast cancer, a set of features was selected to compose the statistical models. Nonetheless, it was observed that a minimal number of features (two features per model, data not shown) would be enough to provide sensitive and accurate classification models, exemplifying the highly discriminant power of lipids for plasma breast cancer differentiation.

Among the molecular contributors for plasma sample differentiation, LysoPC, glycerophospholipids, and triglycerides (TG) were found to be the most important. Indeed, dysregulation of lipid metabolism in tumor cells is known as a hallmark related to the tumors’ opportunistic modes of nutrient acquisition [44]. Other studies reported alterations in the abundance of lysoPC among healthy and breast cancer samples which proved to be consistent with both our models [45,46]. As reported by Taylor et al. [47], LysoPC(16:0) and LysoPC(18:0) are the most abundant types of LysoPC in plasma and their decreased level in breast cancer samples could be associated with an activated inflammatory status and a higher metabolism rate in breast cancer cells. LysoPC are derived from the turnover of PC in circulation, as products of lysophospholipase enzymes such as phospholipase A_2_ (PLA_2_). Yamashita et al. [48] and Yarla et al. [49] reported highly elevated PLA_2_ levels in patients with various malignant tumors, especially in breast cancer. Overexpression or enhanced activity of PLA_2_ is expected to increase LysoPC levels, and this observation could also explain the relative increase in some PC levels observed for healthy plasma samples. Qiu et al. [41] also reported decreased levels of LysoPC in breast plasma samples when compared to healthy samples. Concerning the TG, their higher abundance in cancer plasma may be related to an increased *de novo* lipogenesis. TG are precursors for the synthesis of phospholipids and are also an independent source for fatty acid oxidation. These key processes supply energy and membrane lipids for the accelerated cell proliferation required in tumorigenesis [50,51]. Interestingly, some tocotrienol-related metabolites (analogs of vitamin E) were found in higher abundance in the cancer patient’s plasma. This fact could be related to the usual supplementation of tocotrienols in breast cancer treatment, as these compounds are claimed to suppress the growth of tumors cells [52].

### 3.3. Correspondence of Biomarkers Between Tissue and Plasma

Tissue-specific biomarkers previously reported for breast cancer detection were investigated according to their occurrence in plasma. More than half of tissue biomarkers could be found in plasma. This fact reflects how tissue injury may affect peripheral blood composition [53]. Interestingly, none of the tissue-biomarkers had a significant value for plasma differentiation. We believe that this could be related to two different factors: the dilution of specific tissue biomarkers in the peripheral blood and the different extraction methods applied to plasma and tissue samples.

Tissue-specific lipids detected by DESI-MSI, even when observed in plasma samples, were not predictive for plasma differentiation. This may reflect how specific tissue lipids reach the bloodstream and how diluted they are among other non-specific molecules. Although tissue injury may release specific biomarkers in the bloodstream, the most abundant circulating biomarkers could be secondary products of the injured metabolism. For example, the increased levels of LysoPC found in plasma samples corroborates an increased releasing of arachidonic acid, FA(20:4), also due to PLA_2_ action. The increased abundance of FA(20:4) was noticed as a marker for tumor region over the tissue. However, FA(20:4) was not directly found as a highly discriminant ion for plasma samples, although this molecule is among those detected and identified in plasma extracts.

Moreover, whereas plasma samples were analyzed after exhaustive solvent extraction (i.e., liquid-liquid extraction), tissue samples were not exhaustively extracted and had only their most abundant superficial molecules desorbed/ionized by the charged droplets of the DESI-MSI spray plume. Besides that, the matrix effect in DESI-MSI analysis must be considered. Molecules that would be suppressed by other more abundant components in DESI-MS analysis may be separated in time over a chromatographic column. This results in their proper ionization, detection, and further recognition as significant components in statistical models.

In summary, our study has brought to the attention of the scientific community a comparison of the molecular signatures of breast cancer found directly over tumor tissue by DESI-MSI with those gathered from plasma extracts of the same breast cancer patients by LC-MS. As supported by the literature [4,5,6,7,8,9,28,29,30,31,32], both plasma lipid profiles detected by LC-MS and tissue lipid signatures detected by DESI-MSI could be used for diagnostic purposes. However, we have shown for the first time, that each one of the studied matrices has its specific molecular signatures and the interposition of these signatures was not observed across different sample types (i.e., blood or tissue). Although indicators of tissue injury do not prevail as biomarkers in peripheral blood, some of them could be found in plasma samples demonstrating relevant sensitivity and accuracy of the LC-MS method. Our study also enabled the testing of the classification model described in our previous findings [28] for the analysis of an independent sample set, comprising special type carcinomas, in an inter-laboratory experiment. Special type carcinomas had not been previously used for the building of this classification model and the achievement of 100% specificity/sensitivity rates showcases the discriminatory power of the proposed methodology. These results reinforce DESI-MSI as a robust technique to be used for breast cancer diagnosis including the correct classification of special type carcinomas according to their cancer status, and ER/PR status with remarkable specificity, sensitivity, and accuracy.

## 4. Materials and Methods 

### 4.1. Subjects and Ethical Consent

**Figure 5:**
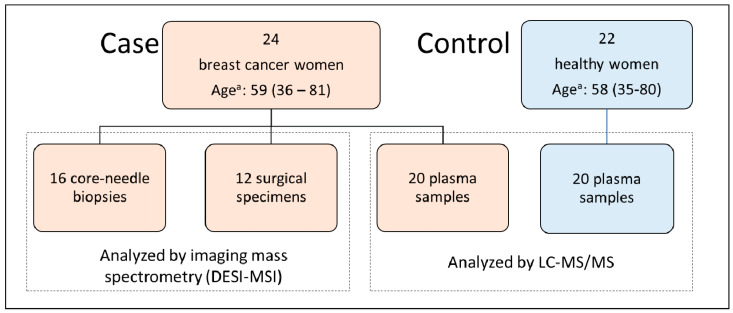
Distribution of samples over case and control groups and the technique of choice employed in each subset: Desorption-Electrospray-Ionization—Mass Spectrometry Imaging (DESI-MSI) for tissue samples and Liquid Chromatography-Tandem Mass Spectrometry (LC-MS/MS) for plasma samples. ^a^ Age is expressed as medium age (range).

The case group comprised 24 women with a confirmed diagnosis for breast cancer. The women had their EDTA-blood samples and/or core-needle biopsies and/or surgical specimens collected. Detailed information about the type of sample collected for each subject is described in Supplementary Table S1. Since not every woman presented the three types of samples, this study comprised the collection of 20 EDTA-blood samples, 16 biopsies, and 12 surgical specimens, resulting in 28 tissue samples (Figure 5). All the recruitment was done during their attendance at the Department of Gynecological and Breast Oncology, Women’s Hospital (CAISM), at the State University of Campinas (UNICAMP), Campinas, São Paulo, Brazil. The procedures were carried out according to the Helsinki Declaration and written informed consent was obtained from each study participant (CAAE # 69699717.0.0000.5404, 09/05/2017, CEP-UNICAMP). The control group comprised 22 healthy women with no evidence of any personal or family story of breast cancer, who donated their EDTA-blood samples under the same collection protocol and served to provide a representative group of the general population that seeks medical assistance in this region. Written informed consent was also obtained from the control women (CAAE # 25222619.4.0000.5514, 13/12/2019, CEP-USF). Table 3 summarizes the clinicopathologic characteristics of women from the breast cancer group.

Estrogen receptor status, progesterone receptor status and HER2 (human-epidermal-growth-factor-receptor-2) receptors status referred to tissue samples. Special Types: pleomorphic lobular breast carcinoma (N = 1), mixed invasive ductal carcinoma/squamous/metaplastic breast carcinoma (N = 1), mucinous colloid breast carcinoma (N = 4), papillary breast carcinoma (N = 1), apocrine breast carcinoma with signet ring (N = 1).

### 4.2. Tissue Samples Analyzed by DESI-MSI

Twenty-eight human breast tissue samples from fragments of core needle biopsy (N = 16) and surgical specimens (N = 12) were collected from women undergoing mastectomy or quadrantectomy as part of their cancer diagnosis and treatment in the Department of Gynecological and Breast Oncology, Women’s Hospital (CAISM) and were later submitted to DESI-MSI analysis. For that, immediately after the removal of the surgical specimen, the tissue was macroscopically assessed, and the tumor area was identified. The presence of tumor was later confirmed through histopathology by an expert pathologist. Surgical specimens and biopsy fragments were snap-frozen using liquid nitrogen within a maximum of 4 h after the surgical removal. The samples were then stored at −80 °C until they were sectioned for DESI-MSI. Tissue samples were sectioned at 16 μm thick sections using a CryoStarTM NX50 cryostat (Thermo Scientific, San Jose, CA, USA) and stored in a −80 °C freezer.

### 4.3. DESI-MSI Experiments

A 2D Omni Spray DESI imaging platform (Prosolia Inc., Indianapolis, IN) coupled to a Q-Exactive Orbitrap (Thermo Fisher Scientific, San Jose, CA) was used for tissue imaging. A lab-built sprayer was adapted to the commercial Omni Spray DESI imaging stage and a solution of dimethylformamide/acetonitrile (DMF/ACN) 1:1 (*v*/*v*) was sprayed at a flow rate of 1.5 μL.min^−1^. The samples were screened in negative-ion mode over the *m/z* range of 100–1200. The resolving power of 70,000 (at *m/z* 400) was used. The S-lens RF level was set to 100, the spatial resolution of 200 μm was used and the nitrogen gas pressure of the DESI source was 150 psi.

### 4.4. Plasma Samples Analyzed by LC-MS

Plasma samples were obtained from collected EDTA-bloods which were centrifuged up to 2 h after the collection time and then frozen until the time of extraction at −80 °C. Before the extraction protocol, quality control samples were prepared by pooling together an aliquot of each sample from both groups. This pool was further spliced into different microtubes and submitted to sample extraction concomitantly with the other samples. After the extraction, all the samples were submitted to LC-MS analysis using electrospray ionization (ESI) in either positive or negative ion modes using an ACQUITY H-class liquid chromatograph coupled to XEVO-G2XS QTOF (Waters) mass spectrometer.

#### 4.4.1. Lipid Extraction

Plasma samples (150 μL) were extracted with the addition of 600 μL of a CHCl_3_:MeOH solution (2:1, *v*/*v*). Afterward, vortex (30 s) and centrifugation (12,000 RPM, 5 min, 4 °C) were carried out and 450 μL of the bottom organic layer were collected and dried under nitrogen flow. Dried samples were stored at −20 °C until the analysis. For analysis, samples were reconstituted in 1 mL of a solution of isopropanol (IPA)/ACN/water (2:1:1, *v*/*v*/*v*).

#### 4.4.2. LC-MS Analysis

An ACQUITY UPLC connected to a XEVO-G2XS quadrupole time-of-flight (QTOF) mass spectrometer (Waters, Manchester, UK) equipped with an electrospray ion source was used. Liquid chromatography was performed using an Acquity UPLC CSH C18 column (2.1 × 100 mm, 1.7 μm, Waters). Mobile phase A was composed of a solution of 10 mM ammonium formate with 0.1% formic acid in ACN/water (60:40, *v*/*v*), while mobile phase B was composed of a solution of 10 mM ammonium formate with 0.1% formic acid in IPA/ACN (90:10, *v*/*v*). The flow rate was 0.4 mL min^−1^. The column was initially eluted with 40% B, increasing to 43% B over 2 min and subsequently to 50% within 0.1 min. Over the next 3.9 min, the gradient was further ramped to 54% B, and then to 70% of B in 0.1 min. In the final part of the gradient, the amount of B was increased to 99% over 1.9 min. Solution B finally returned to 40% in 0.1 min, and the column was equilibrated for 1.9 min before the next injection. The total run time was 10 min. The injection volume was 1 μL. Positive and negative ion modes were recorded (separately) and the instrument was operated in MS^E^ mode in the *m/z* range of 50–2000, with an acquisition time of 1 s per scan. Other parameters were as follows: source temperature = 120 °C, desolvation temperature = 600 °C, desolvation gas flow = 800 L·h^−1^, capillary voltage = 2.0 kV(+)/1.5 kV(–), cone voltage = 30 V. Leucine encephalin (molecular weight = 555.62; 200 pg·μL^−1^ in 1:1 ACN:H_2_O) was used as a lock mass for accurate mass measurements, and a 0.5 mM sodium formate solution was used for calibration. Samples were randomly analyzed and quality control samples were injected every ten injections.

#### 4.4.3. Data Extraction

LC-MS raw files were processed using Progenesis QI 2.0 software (Nonlinear Dynamics, Newcastle, UK), which enabled raw data import, selection of possible adducts, peak alignment, deconvolution, and compound identification based on MS^E^ experiments. Progenesis QI generates a table of the ions labeled according to their nominal masses and retention times as a function of their intensity for each sample. Examples of the chromatograms, raw spectra, and feature identification are shown in Figures S1–S3, for the positive and negative ion modes.

### 4.5. Statistical Analysis

For tissue analysis, MS data corresponding to the areas of interest were extracted from the ion images using the MSiReader software [54]. Data preprocessing was performed following Porcari et al. [28]. The previously generated logistic regression model with Lasso (least absolute shrinkage and selection operator) regularization was used to predict tissue samples according to the presence of breast cancer, estrogen receptor (ER) status, and progesterone receptor (PR) status.

For plasma analysis, the list of extracted ions chromatograms per retention time was uploaded to the MetaboAnalyst web platform (http://www.metaboanalyst.ca). Data were normalized by sum and auto-scaled. Ions detected in at least 10% of the samples were held for analysis. Comparisons were performed of the case against control groups. For the unsupervised analysis, principal component analysis (PCA) was used. For the supervised analysis through support vector machine (SVM), data were divided into a training set (70% of samples) and a validation set (30% of samples). The training set was composed of plasma of 14 breast cancer women and plasma of 15 healthy women, whereas the validation set was composed of plasma of 6 breast cancer women and 7 healthy women. The biomarker analysis module provided by the Metaboanalyst web platform was used and data was loaded in the form of a table containing the list of extracted ions chromatograms per retention time and the group label for 70% of the samples. Thirty percent of the samples had no group label (test samples). The same parameters used for PCA were chosen for filtering, normalization, and scaling of the data. The classification method was linear SVM, whereas the selected feature ranking method was built-in SVM. To evaluate the test set, the top 10 features with the highest area under the ROC curve (AUC) value were selected to compose the final SVM model, which was used to classify and provide the medium probability of correct classification for each test sample.

To investigate whether the biomarkers validated as discriminatory for tissue analysis could also be predictive in plasma, the tissue biomarkers detected in plasma were used to build a PCA model. In that way, their discriminatory power regarding plasma samples of case and control groups was evaluated.

## Figures and Tables

**Table 1:** Discriminant ions selected by Supporting Vector Machine (SVM) models (positive and negative ion mode) as significant contributors to the molecular classification of plasma from healthy and cancer women.

Measured *m/z*	Ion Mode	Species	Lipid Assignment	Proposed Formula	Exact *m/z*	Mass Error (ppm)
Characteristic of healthy plasma samples						
496.340	+	[M + H]^+^	LysoPC(16:0)	C_24_H_51_NO_7_P	496.340	0.0
524.371	+	[M + H]^+^	LysoPC(18:0)	C_26_H_55_NO_7_P	524.372	1.9
782.569	+	[M + H]^+^	PC(40:4)	C_44_H_81_NO_8_P	782.570	1.3
810.600	+	[M + H]^+^	PC(38:4)	C_46_H_85_NO_8_P	810.601	1.2
540.330	−	[M + FA − H]^−^	LysoPC(16:0)	C_25_H_51_NO_9_P	540.330	0.0
568.361	−	[M + FA − H]^−^	LysoPC(18:0)	C_27_H_55_NO_9_P	568.361	0.0
588.330	−	[M + FA − H]^−^	LysoPC (20:4)	C_29_H_51_NO_9_P	588.330	0.0
566.346	−	[M + FA − H]^−^	LysoPC(18:1)	C_27_H_53_NO_9_P	566.346	0.0
Characteristic of cancer plasma samples						
786.600	+	[M + H]^+^	PC(36:2)	C_44_H_85_NO_8_P	786.601	1.3
796.738	+	[M + NH_4_]^+^	TG (46:0)	C_49_H_98_NO_6_	796.739	1.3
758.570	+	[M + H]^+^	PC(34:2)	C_42_H_81_NO_8_P	758.570	0.0
824.770	+	[M + NH_4_]^+^	TG(48:0)	C_51_H_102_NO_6_	824.771	1.2
407.294	−	[M − H_2_O − H]^−^	13′-Hydroxy-gamma-tocotrienol	C_28_H_39_O_2_	407.295	2.5
409.310	−	[M − H]^−^	gamma-tocotrienol	C_28_H_41_O_2_	409.311	2.4
802.559	−	[M + FA − H]^−^	PC(34:2)/PE-Nme(36:2)	C_43_H_81_NO_10_P	802.560	1.2
830.590	−	[M + FA − H]^−^	PC(36:2)	C_45_H_85_NO_10_P	830.591	1.2
776.544	−	[M + FA − H]^−^	PC(32:1)	C_41_H_79_NO_10_P	776.544	0.0

*m/z:* mass-to-charge ratio; ppm: parts per million; LysoPC: Lysophosphatidylcholine; PC: phosphatidylcholine; TG: triglyceride; PE-Nme: methylphosphatidylethanolamine. Lipid species are described by the numbers of fatty acid chain carbons and double bonds.

**Table 2:** Tissue biomarkers found by DESI-MSI (Desorption-Electrospray-Ionization—Mass Spectrometry Imaging) and their occurrence in plasma samples of breast cancer patients analyzed by LC-MS/MS (Liquid Chromatography—tandem Mass Spectrometry).

Tissue Biomarkers for No Special Type (NST) Ductal Carcinoma of the Breast [28]	Prevalence in Plasma Samples of Breast Carcinoma (NST and Special Type) Patients According to LC-MS/MS Results
PS(34:1); PE(38:4); PS(38:4); PI(34:1); PS(40:4); PI(36:2); PI(38:3); PE(36:2); PE(O-38:6); PE(O-38:5); PS(36:2); PS(36:1); PC(34:2); PC(34:1); PS(38:1); PI(34:0); PI(38:4)	Yes
PG(34:1); PG(36:2); PG(40:7); PS(O-41:0); Cer(t42:1); CL(72:8); CL(72:7); PA(38:2); PS(O-33:0); PE(O-38:4); PG(36:4); PS(P-36:2); PE(39:5); TG(52:3)	No

PS: glycerophosphoserine, PE: glycerophosphoethanolamine; PI: glycerophosphoinositol; PC: glycerophosphocholine; PG: glycerophosphoglycerol; Cer: ceramide; CL: cardiolipin; PA: phosphatidic acid; TG: triacylglycerol. Lipid species are described by the numbers of fatty acid chain carbons and double bonds.

**Table 3:** Clinicopathologic characteristics of women with breast cancer.

Characteristics	Patients, N	Median Age (Range)
Core needle biopsy	16	60 (37–80)
Surgical specimen	12	61 (36–81)
Core needle biopsy + surgical specimen	5	63 (37–80)
Plasma	20	58 (36–81)
*Tumor type*		
Ductal NST (no special type)	16	56 (36–81)
Special Types	8	65 (37–80)
*Tumor stage*		
I	10	57 (43–77)
II	8	59 (36–81)
III	3	64 (37–80)
IV	3	61 (42–75)
*Estrogen receptor status*		
Positive	20	58 (36–81)
Negative	4	65 (42–77)
*Progesterone receptor status*		
Positive	16	56 (36–81)
Negative	8	65 (42–80)
*HER2 receptor status*		
Positive	6	47 (36–67)
Negative	18	63 (38–81)

## Supplementary Materials

Supplementary Materials can be found at https://www.mdpi.com/1422-0067/21/10/3611/s1.

## Abbreviations

AUC	Area under the (ROC) curve
CAAE	Brazilian certificate of ethical appreciation approval
CAISM-UNICAMP	Center of integrated assistance to women’s health of the University of Campinas
Cer	Ceramide
CL	Cardiolipin
CN:DB	Numbers of fatty acid chain carbons and double bonds in lipid species
DC	Ductal carcinoma
DCIS	In situ ductal carcinoma
DESI-MSI	Desorption-Electrospray-Ionization—Mass Spectrometry
ER	Estrogen receptor
FA	Fatty acid
H&E	Hematoxylin and eosin staining
HER2	Human-epidermal-growth-factor-receptor-2
HR	Hormone receptor status
IDC	Invasive ductal carcinoma of the breast
Lasso	Least absolute shrinkage and selection operator
LC-MS/MS	Liquid chromatography coupled to tandem mass spectrometry
LC-MS	Liquid chromatography coupled to mass spectrometry
LysoPC	Lysophosphatidylcholine
*m/z*	Mass-to-charge ratio
MS	Mass Spectrometry
MS^E^	MS data-independent acquisition
MSI	Mass Spectrometry Imaging
NST	no special type carcinoma of the breast
NPV	Negative predictive value
PA	Phosphatidic acid
PC	Glycerophosphocholine
PC1	Principal component 1
PC2	Principal component 2
PCA	Principal Component Analysis
PE	Glycerophosphoethanolamine
PE	Phosphatidylethanolamine
PE-Nme	Methylphosphatidylethanolamine
PG	Glycerophosphoglycerol
PI	Glycerophosphoinositol
ppm	parts per million
PPV	Positive predictive value
PR	Progesterone receptor
PS	Glycerophosphoserine
QC	Quality control sample
ROC	Receiver operating-characteristic (curves)
SVM	Support Vector Machine
TG	Triacylglycerol or Triglyceride
TIC	Total Ion Current

## References

[1] Gogiashvili M., Nowacki J., Hergenröder R., Hengstler J.G., Lambert J., Edlund K. (2019). HR-MAS NMR Based Quantitative Metabolomics in Breast Cancer. Metabolites.

[2] Hart C.D., Tenori L., Luchinat C., Di Leo A., Stearns V. (2016). Metabolomics in Breast Cancer: Current Status and Perspectives. Novel Biomarkers in the Continuum of Breast Cancer.

[3] Wishart D.S. (2016). Emerging applications of metabolomics in drug discovery and precision medicine. Nat. Rev. Drug Discov..

[4] Jasbi P., Wang D., Cheng S.L., Fei Q., Cui J.Y., Liu L. (2019). Breast cancer detection using targeted plasma metabolomics. J. Chromatogr. B. Analyt. Technol. Biomed. Life Sci..

[5] Huang M., Li H.-Y., Liao H.-W., Lin C.-H., Wang C.-Y., Kuo W.-H., Kuo C.-H. (2019). Using post-column infused internal standard assisted quantitative metabolomics for establishing prediction models for breast cancer detection. Rapid Commun. Mass Spectrom..

[6] Lecuyer L., Dalle C., Lyan B., Demidem A., Rossary A., Vasson M.P., Petera M., Lagree M., Ferreira T., Centeno D. (2019). Plasma Metabolomic Signatures Associated with Long-term Breast Cancer Risk in the SU.VI.MAX Prospective Cohort. Cancer Epidemiol. Biomarkers Prev..

[7] Zhang Q., Xu H., Liu R., Gao P., Yang X., Jin W. (2019). A Novel Strategy for Targeted Lipidomics Based on LC-Tandem-MS Parameters Prediction, Quantification, and Multiple Statistical Data Mining: Evaluation of Lysophosphatidylcholines as Potential Cancer Biomarkers. Anal. Chem..

[8] Cala M.P., Aldana J., Medina J., Sanchez J., Guio J., Wist J. (2018). Multiplatform plasma metabolic and lipid fingerprinting of breast cancer: A pilot control-case study in Colombian Hispanic women. PLoS ONE.

[9] Zhang Q., Liu R., Xu H., Yang X., Zhang Y., Wang Q., Gao P., Bi K., Han T., Li Q. (2020). Multifunctional isotopic standards based steroidomics strategy: Exploration of cancer screening model. J. Chromatogr. A.

[10] Luo X., Yu H., Song Y., Sun T. (2019). Integration of metabolomic and transcriptomic data reveals metabolic pathway alteration in breast cancer and impact of related signature on survival. J. Cell Physiol..

[11] Park J., Shin Y., Kim T.H., Kim D.H., Lee A. (2019). Plasma metabolites as possible biomarkers for diagnosis of breast cancer. PLoS ONE.

[12] Chen X., Chen H., Dai M., Ai J., Li Y., Mahon B., Dai S., Deng Y. (2016). Plasma lipidomics profiling identified lipid biomarkers in distinguishing early-stage breast cancer from benign lesions. Oncotarget.

[13] Yamashita Y., Nishiumi S., Kono S., Takao S., Azuma T., Yoshida M. (2017). Differences in elongation of very long chain fatty acids and fatty acid metabolism between triple-negative and hormone receptor-positive breast cancer. BMC Cancer.

[14] Li L., Zheng X., Zhou Q., Villanueva N., Nian W., Liu X., Huan T. (2020). Metabolomics-Based Discovery of Molecular Signatures for Triple Negative Breast Cancer in Asian Female Population. Sci. Rep..

[15] Lin X., Xu R., Mao S., Zhang Y., Dai Y., Guo Q., Song X., Zhang Q., Li L., Chen Q. (2019). Metabolic biomarker signature for predicting the effect of neoadjuvant chemotherapy of breast cancer. Ann. Transl. Med..

[16] Silva C., Perestrelo R., Silva P., Tomás H., Câmara J.S. (2019). Breast Cancer Metabolomics: From Analytical Platforms to Multivariate Data Analysis. A Review. Metabolites.

[17] Cardoso M.R., Santos J.C., Ribeiro M.L., Talarico M.C.R., Viana L.R., Derchain S.F.M. (2018). A Metabolomic Approach to Predict Breast Cancer Behavior and Chemotherapy Response. Int. J. Mol. Sci..

[18] McCartney A., Vignoli A., Biganzoli L., Love R., Tenori L., Luchinat C., Di Leo A. (2018). Metabolomics in breast cancer: A decade in review. Cancer Treat. Rev..

[19] Leung F., Eberlin L.S., Schwamborn K., Heeren R.M., Winograd N., Cooks R.G. (2019). Mass Spectrometry-Based Tissue Imaging: The Next Frontier in Clinical Diagnostics?. Clin. Chem..

[20] Perez C.J., Bagga A.K., Prova S.S., Taemeh M.Y., Ifa D.R. (2019). Review and perspectives on the applications of mass spectrometry imaging under ambient conditions. Rapid Commun. Mass Spectrom..

[21] Feider C.L., Krieger A.C., Dehoog R.J., Eberlin L.S. (2019). Ambient ionization mass spectrometry: Recent developments and applications. Anal. Chem..

[22] Ifa D.R., Eberlin L.S. (2016). Ambient Ionization Mass Spectrometry for Cancer Diagnosis and Surgical Margin Evaluation. Clin. Chem..

[23] Woolman M., Zarrine-Afsar A. (2018). Platforms for rapid cancer characterization by ambient mass spectrometry: Advancements, challenges, and opportunities for improvement towards intrasurgical use. Analyst.

[24] Jarmusch A.K., Pirro V., Baird Z., Hattab E.M., Cohen-Gadol A.A., Cooks R.G. (2016). Lipid, and metabolite profiles of human brain tumors by desorption electrospray ionization-MS. Proc. Natl. Acad Sci. USA.

[25] Eberlin L.S., Ifa D.R., Wu C., Cooks R.G. (2010). Three-Dimensional Vizualization of Mouse Brain by Lipid Analysis Using Ambient Ionization Mass Spectrometry. Angew. Chem. Int. Edit..

[26] Eberlin L.S., Gabay M., Fan A.C., Gouw A.M., Tibshirani R.J., Felsher D.W., Zare R.N. (2014). Alteration of the lipid profile in lymphomas induced by MYC overexpression. Proc. Natl. Acad Sci. USA.

[27] Woolman M., Tata A., Dara D., Meens J., D’Arcangelo E., Perez C.J., Saiyara Prova S., Bluemke E., Ginsberg H.J., Ifa D. (2017). Rapid determination of the tumour stroma ratio in squamous cell carcinomas with desorption electrospray ionization mass spectrometry (DESI-MS): A proof-of-concept demonstration. Analyst.

[28] Porcari A.M., Zhang J., Garza K.Y., Rodrigues-Peres R.M., Lin J.Q., Young J.H., Tibshirani R., Nagi C., Paiva G.P., Carter S.A. (2018). Multicenter Study Using Desorption-Electrospray-Ionization-Mass-Spectrometry Imaging for Breast-Cancer Diagnosis. Anal. Chem..

[29] Calligaris D., Caragacianu D., Liu X., Norton I., Thompson C.J., Richardson A.L., Golshan M., Easterling M.L., Santagata S., Dillon D.A. (2014). Application of desorption electrospray ionization mass spectrometry imaging in breast cancer margin analysis. Proc. Natl. Acad Sci. USA.

[30] Guenther S., Muirhead L.J., Golf O., Strittmatter N., Ramakrishnan R., Goldin R.D., Jones E.A., Veselkov K., Darzi A., Takáts Z. (2015). Spatially Resolved Metabolic Phenotyping of Breast Cancer by Desorption Electrospray Ionization Mass Spectrometry. Cancer Res..

[31] Tata A., Gribble A., Ventura M., Ganguly M., Bluemke E., Ginsberg H.J., Jaffray D.A., Ifa D.R., Vitkin A., Zarrine-Afsar A. (2016). Wide-field tissue polarimetry allows efficient localized mass spectrometry imaging of biological tissues. Chem. Sci..

[32] Woolman M., Tata A., Bluemke E., Dara D., Ginsberg H.J., Zarrine-Afsar A. (2017). An Assessment of the Utility of Tissue Smears in Rapid Cancer Profiling with Desorption Electrospray Ionization Mass Spectrometry (DESI-MS). J. Am. Soc. Mass Spectrom.

[33] Tata A., Woolman M., Ventura M., Bernards N., Ganguly M., Gribble A., Shrestha B., Bluemke E., Ginsberg H.J., Vitkin A. (2016). Rapid Detection of Necrosis in Breast Cancer with Desorption Electrospray Ionization Mass Spectrometry. Sci. Rep..

[34] Santoro A.L., Drummond R.D., Silva I.T., Ferreira S.S., Juliano L., Vendramini P.H., Lemos M.B.D.C., Eberlin M.N., De Andrade V.P. (2020). In Situ DESI-MSI Lipidomic Profiles of Breast Cancer Molecular Subtypes and Precursor Lesions. Cancer Res..

[35] Kertesz V., Van Berkel G.J., Vavrek M., Koeplinger K.A., Schneider B.B., Covey T.R. (2008). Comparison of drug distribution images from whole-body thin tissue sections obtained using desorption electrospray ionization tandem mass spectrometry and autoradiography. Anal. Chem.

[36] Wiseman J.M., Ifa D.R., Zhu Y., Kissinger C.B., Manicke N.E., Kissinger P.T. (2008). Desorption electrospray ionization mass spectrometry: Imaging drugs and metabolites in tissues. Proc. Natl. Acad Sci. USA.

[37] Vismeh R., Waldon D.J., Teffera Y., Zhao Z. (2012). Localization, and quantification of drugs in animal tissues by use of desorption electrospray ionization mass spectrometry imaging. Anal. Chem..

[38] Tata A., Perez C., Campos M.L., Bayfield M.A., Eberlin M.N., Ifa D.R. (2015). Imprint Desorption Electrospray Ionization Mass Spectrometry Imaging for Monitoring Secondary Metabolites Production during Antagonistic Interaction of Fungi. Anal. Chem..

[39] Abbassi-Ghadi N., Jones E.A., Romero M.G., Golf O., Kumar S., Huang J., Kudo H., Goldin R.D., Hanna G.B., Takáts Z. (2015). A Comparison of DESI-MS and LC-MS for the Lipidomic Profiling of Human Cancer Tissue. J. Am. Soc. Mass Spectrom..

[40] Gromski P.S., Muhamadali H., Ellis D.I., Xu Y., Correa E., Turner M.L., Goodacre R. (2015). A tutorial review: Metabolomics and partial least squares-discriminant analysis—a marriage of convenience or a shotgun wedding. Anal. Chim. Acta.

[41] Qiu Y., Zhou B., Su M., Baxter S., Zheng X., Zhao X., Yen Y., Jia W. (2013). Mass Spectrometry-Based Quantitative Metabolomics Revealed a Distinct Lipid Profile in Breast Cancer Patients. Int. J. Mol. Sci..

[42] Jiang N., Zhang G., Pan L., Yan C., Zhang L., Weng Y., Wang W., Chen X., Yang G. (2017). Potential plasma lipid biomarkers in early-stage breast cancer. Biotechnol. Lett..

[43] Goodacre R., Vaidyanathan S., Dunn W.B., Harrigan G.G., Kell D.B. (2004). Metabolomics by numbers: Acquiring and understanding global metabolite data. Trends Biotechnol..

[44] Pavlova N.N., Thompson C.B. (2016). The Emerging Hallmarks of Cancer Metabolism. Cell Metab..

[45] Brglez V., Pucer A., Pungercar J., Lambeau G., Petan T. (2014). Secreted phospholipases A (2)are differentially expressed and epigenetically silenced in human breast cancer cells. Biochem. Biophys Res. Commun..

[46] Iorio E., Caramujo M.J., Cecchetti S., Spadaro F., Carpinelli G., Canese R., Podo F. (2016). Key Players in Choline Metabolic Reprograming in Triple-Negative Breast Cancer. Front. Oncol..

[47] Taylor L.A., Arends J., Hodina A.K., Unger C., Massing U. (2007). Plasma lyso-phosphatidylcholine concentration is decreased in cancer patients with weight loss and activated inflammatory status. Lipids Heal. Dis..

[48] Yamashita S., Yamashita J., Ogawa M. (1994). Overexpression of group II phospholipase A2 in human breast cancer tissues is closely associated with their malignant potency. Br. J. Cancer.

[49] Yarla N.S., Satyakumar K., Srinivasu D., Dsvkg K., Aliev G., Dharmapuri G. (2015). Phospholipase A2: A Potential Therapeutic Target in Inflammation and Cancer (In silico, In vitro, In vivo and Clinical Approach). J. Cancer Sci. Therapy.

[50] Lofterød T., Mortensen E.S., Nalwoga H., Wilsgaard T., Frydenberg H., Risberg T., Eggen A.E., McTiernan A., Aziz S., Wist E.A. (2018). Impact of pre-diagnostic triglycerides and HDL-cholesterol on breast cancer recurrence and survival by breast cancer subtypes. BMC Cancer.

[51] Zhang F., Du G. (2012). Dysregulated lipid metabolism in cancer. World J. Biol. Chem..

[52] Sailo B.L., Banik K., Padmavathi G., Javadi M., Bordoloi D., Kunnumakkara A.B. (2018). Tocotrienols: The promising analogues of vitamin E for cancer therapeutics. Pharmacol. Res..

[53] Sheth S.A., Iavarone A.T., Liebeskind D.S., Won S.J., Swanson R.A. (2015). Targeted Lipid Profiling Discovers Plasma Biomarkers of Acute Brain Injury. PLoS ONE.

[54] Bokhart M., Nazari M., Garrard K.P., Muddiman D.C. (2017). MSiReader v1.0: Evolving Open-Source Mass Spectrometry Imaging Software for Targeted and Untargeted Analyses. J. Am. Soc. Mass Spectrom..

