# Supplementary material for: Multiplatform Investigation of Plasma and Tissue Lipid Signatures of Breast Cancer Using Mass Spectrometry Tools
Source: Int J Mol Sci. 2020 May 20;21(10):3611. doi: 10.3390/ijms21103611 (PMC7279467; doi:10.3390/ijms21103611)
Supplement: Supplementary file 1 [file ijms-21-03611-s001.pdf]

Multiplatform investigation of plasma and tissue lipid signatures of breast cancer using mass spectrometry tools

Alex Ap. Rosini Silva [1,†], Marcella R. Cardoso [2,†], Luciana Montes Resende [2], John Q. Lin [3], Fernando Guimaraes [2], Geisilene R. Paiva Silva [4], Michael Murgu [5], Denise Gonçalves Priolli [1], Marcos N. Eberlin[6], Alessandra Tata [7], Livia S. Eberlin [3], Sophie F. M. Derchain [2] and Andreia M. Porcari [1,*]

[1] Postgraduate Program of Health Sciences, São Francisco University, Bragança Paulista, SP 12916-900, Brazil; alexrosinisilva@hotmail.com (A.A.R.S.); denise.priolli@usf.edu.br (D.G.P.)

[2] Department of Gynecological and Breast Oncology, Women's Hospital (CAISM), Faculty of Medical Sciences, State University of Campinas (UNICAMP), Campinas, SP 13083-881, Brazil; macardoso86@hotmail.com (M.C.); luciana_mrezende@hotmail.com (L.M.R.); fernando@caism.unicamp.br (F.G.); derchain@fcm.unicamp.br (S.F.M.D.)

[3] Department of Chemistry, The University of Texas at Austin, Austin, TX 78712, United States; john@jqlin.com (J.Q.L.); liviase@utexas.edu (L.S.E.)

[4] Laboratory of Molecular and Investigative Pathology – LAPE, Women's Hospital (CAISM), Faculty of Medical Sciences, State University of Campinas (UNICAMP) – Campinas, SP 13083-881, Brazil; geisi@unicamp.br (G.R.P.S.)

[5] Waters Corporation, São Paulo, SP 13083-970, Brazil; Michael_Murgu@waters.com (M.M.)

[6] Mackenzie Presbyterian University, School of Engineering, São Paulo, SP 01302-907, Brazil; mneberlin@gmail.com

[7] Istituto Zooprofilattico Sperimentale delle Venezie, Laboratorio di Chimica Sperimentale, Viale Fiume 78, Vicenza, Italy; atata@izsvenezie.it

† These authors contributed equally to this work

* Correspondence: andreia.porcari@usf.edu.br Tel.; +55-11-2454-8047

Supplementary information contains:

- A table with the summary of the type of samples collected for each breast cancer woman
- Supplementary Figures

Supplementary Table S1: Summary of the type of samples collected for each breast cancer woman

**Subject #**	**Type of sample**			**Type**
	**plasma**	**biopsy**	**surgical specimen**	**NST[1] / ST[2]**
**1**	yes	yes	yes	ST
**2**	yes	yes	yes	ST
**3**	yes	yes	yes	NST
**4**	yes	yes	yes	NST
**5**	yes	yes	yes	ST
**6**	yes	yes	no	ST
**7**	yes	yes	no	NST
**8**	yes	yes	no	NST
**9**	yes	yes	no	NST
**10**	yes	yes	no	NST
**11**	yes	yes	no	NST
**12**	yes	yes	no	ST
**13**	yes	yes	no	NST
**14**	yes	yes	no	ST
**15**	yes	no	yes	NST
**16**	yes	no	yes	NST
**17**	yes	no	yes	ST
**18**	yes	no	yes	NST
**19**	yes	no	yes	ST
**20**	yes	no	no	NST
**21**	no	yes	no	NST
**22**	no	yes	no	NST
**23**	no	no	yes	NST
**24**	no	no	yes	NST

[1]ST: special type ductal carcinoma of the breast. [2]NST: no special type.

Note: We included 24 women with breast cancer. From them, 20 women had blood samples collected and 23 had cancer breast tissue collected. Among women who had their tissue collected, 11 of them had only biopsy, 7 had only surgical specimens and 5 had biopsy and surgical specimens.

Supplementary Figures

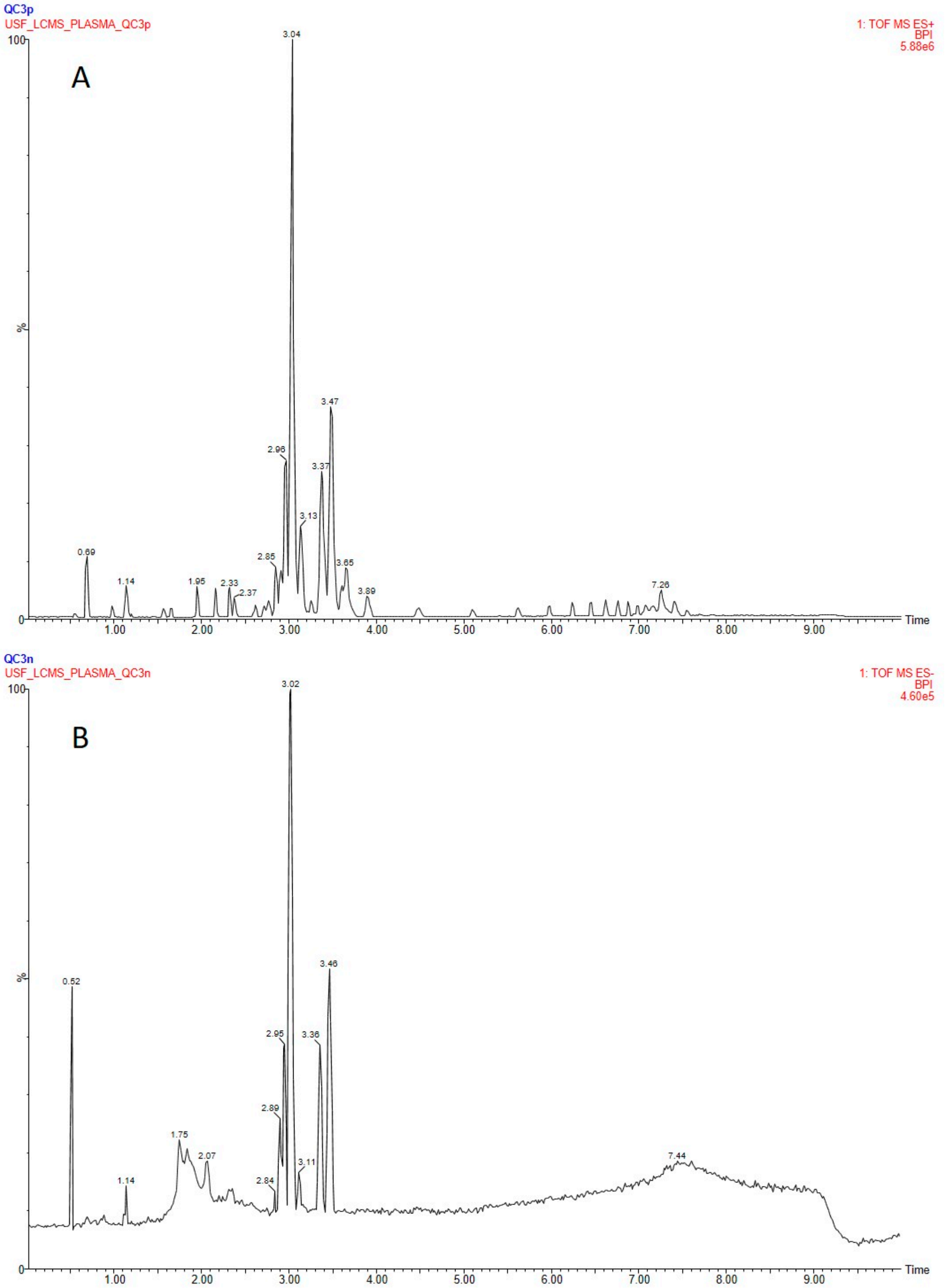

Figure S1: Base peak ion (BPI) chromatograms for a representative quality control (QC) sample acquired in (A) the positive ion mode and (B) the negative ion mode.

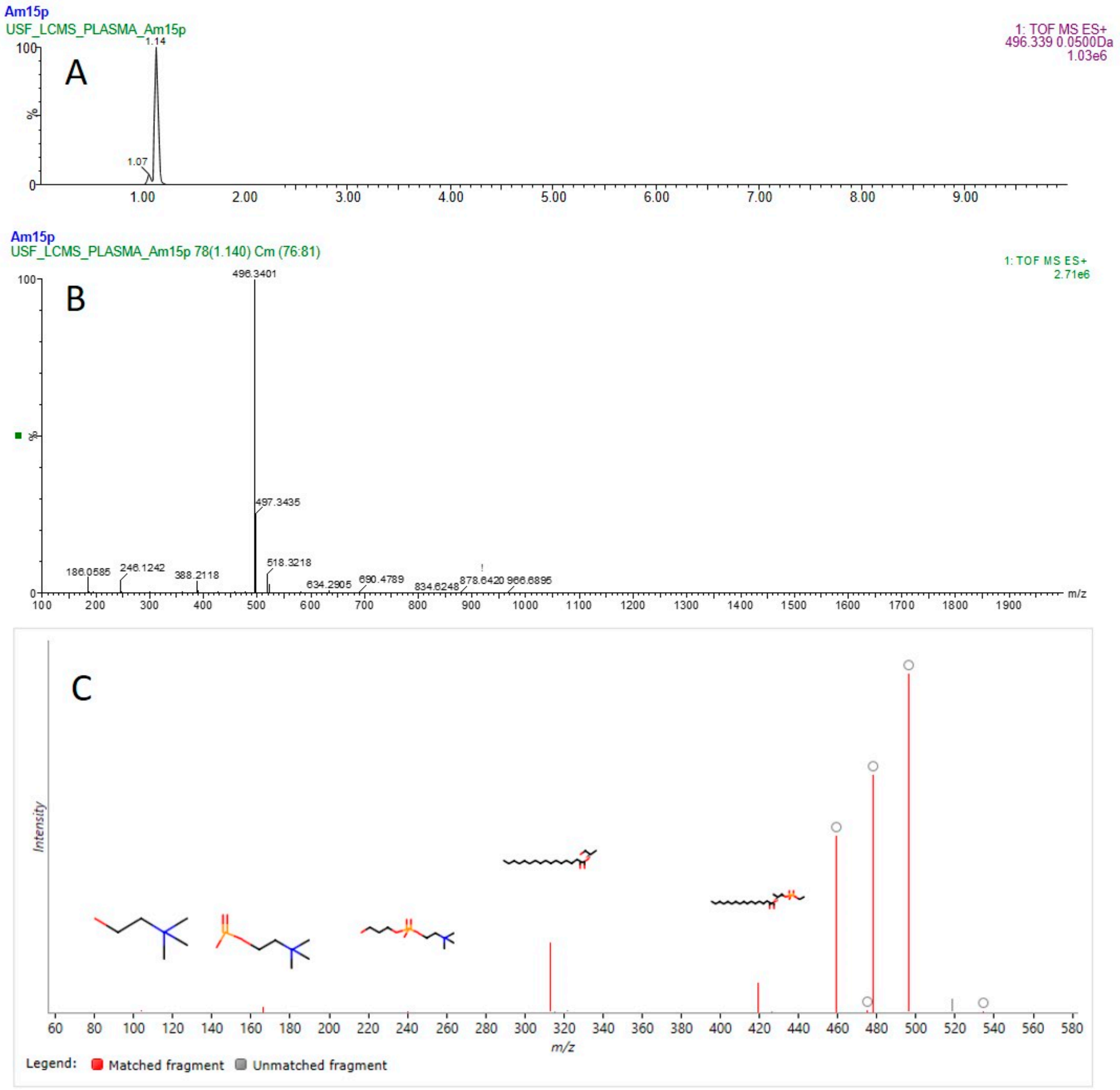

Figure S2: Data for one characteristic ion of healthy plasma samples observed in the positive ion mode. The LysoPC (16:0), observed as the $[M + H]^+$ adduct at *m/z* 496.3399. The extracted ion chromatogram (EIC) is shown in (A). The low-energy mass spectrum is shown in (B). The deconvoluted fragmentation spectrum obtained by Progenesis QI (Waters), showing the identifying fragments is shown in (C), where red signalizes the matched fragments, according to theoretical fragmentation.

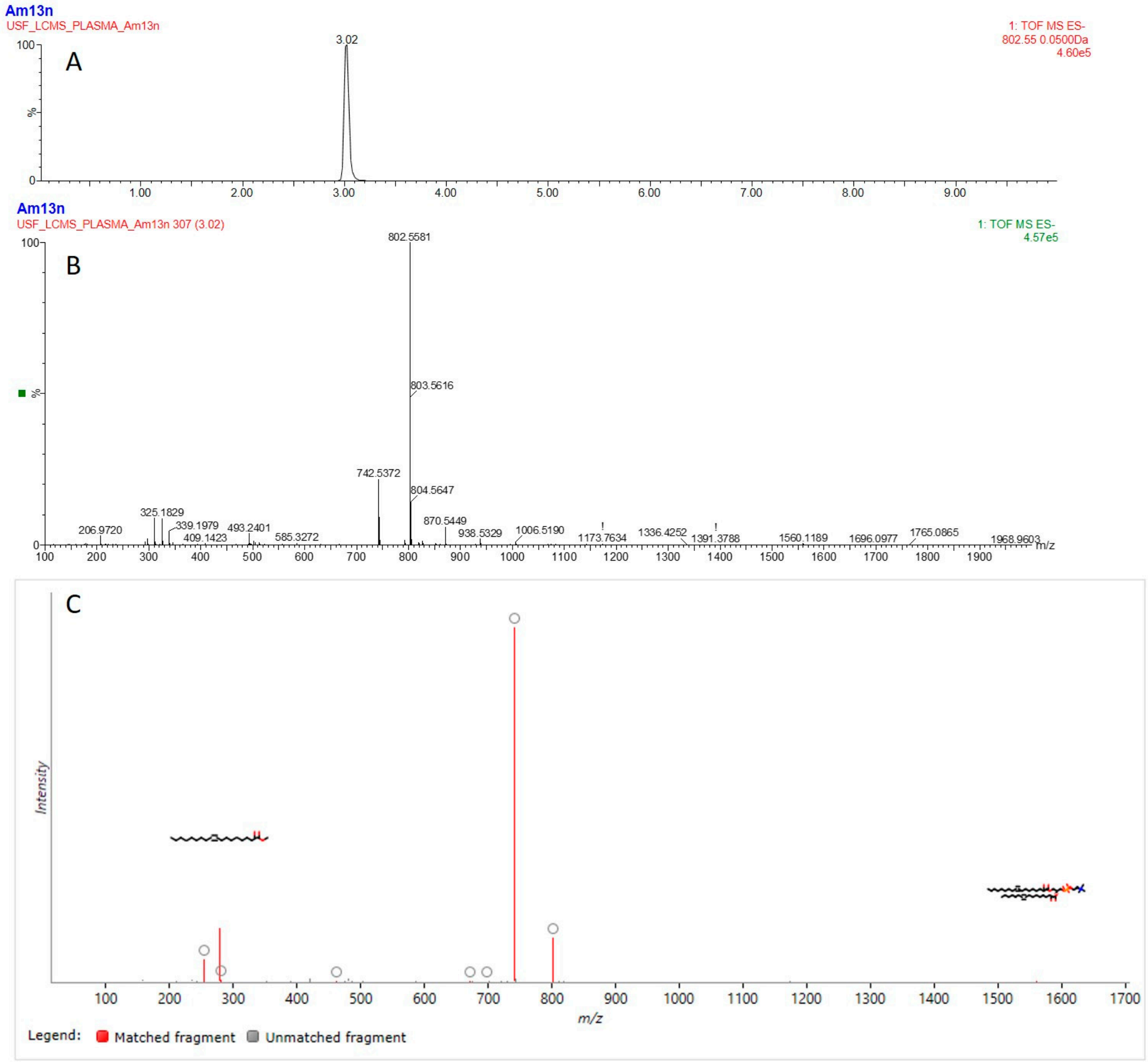

Figure S3: Data for one characteristic ion of cancer plasma samples observed in the negative ion mode. The PC(34:2)/PE-Nme(36:2), observed as the $[M + FA - H]^-$ adduct at *m/z* 802.5593. The extracted ion chromatogram (EIC) is shown in (A). The low-energy mass spectrum is shown in (B). The deconvoluted fragmentation spectrum obtained by Progenesis QI (Waters), showing the identifying fragments is shown in (C), where red signalizes the matched fragments, according to theoretical fragmentation.

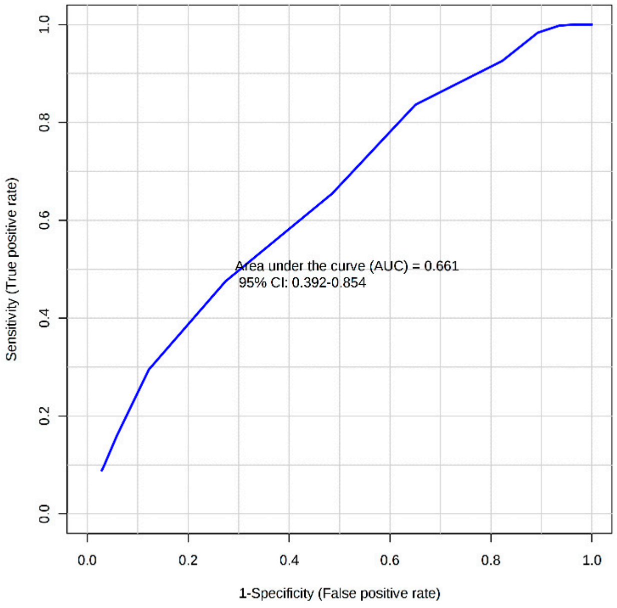

Figure S4: Receiver operator characteristics (ROC) curve for the support vector machine (SVM) model for differentiation among cancer and health status based on plasma lipids listed as molecular signatures for tissue differentiation using imaging mass spectrometry. A near to diagonal ROC curve represents an insufficient discriminatory power for the model. AUC is the area under the ROC curve and CI is the confidence interval.
